# Scenario based traffic optimization in Egypt performance gains through simulation modeling

**DOI:** 10.1038/s41598-026-41535-x

**Published:** 2026-03-28

**Authors:** Nehal Fawzy, M. A. Mohamed, Hanan M. Amer, Mohamed Maher Ata

**Affiliations:** 1 Department of Electronics and Communications Engineering, Faculty of Engineering, Mansoura University, Mansoura, 35516 Egypt; 2 School of Computational Sciences and Artificial Intelligence (CSAI), Zewail City of Science and Technology, October 6 City, Giza Egypt

**Keywords:** Intelligent transportation systems (ITS), Simulation of urban mobility (SUMO), Vehicle emissions (CO, CO_2_, NOx, HC, PMx), Engineering, Civil engineering, Electrical and electronic engineering

## Abstract

Rapid urbanization and the growing number of vehicles in Mansoura, Egypt, have exacerbated traffic congestion, underscoring the need for efficient urban traffic management solutions. This study evaluates traffic control strategies at Umm Kulthum Square using the SUMO (Simulation of Urban Mobility) software. Two scenarios were analyzed: Scenario 1 represents existing traffic conditions, with road geometry and lane counts adjusted to real-world dimensions derived from satellite imagery, while Scenario 2 applies optimized lane connections, turning movements, and traffic signal configurations, including signal locations, phase durations, and signal states. Simulations were conducted for 999 s and 3599 s to evaluate traffic performance, queuing behavior, emissions, fuel consumption, and noise levels. Compared with Scenario 1, Scenario 2 reduced average departure delay from 6.02 to 5.54 s at 999 s and from 32.63 to 7.08 s at 3599 s. Average travel duration decreased from 414.19 to 349.25 s and from 388.60 to 246.15 s, while waiting time was reduced from 139.90 to 100.90 s and from 288.21 to 143.70 s, respectively. The number of completed trips increased from 262 to 317 vehicles at 999 s and from 672 to 715 vehicles at 3599 s. Environmental performance also improved, with notable reductions in CO₂, CO, HC, NOx, and PMx emissions, as well as fuel consumption and noise levels. Additionally, queuing time and queue lengths decreased, indicating smoother traffic flow. These results demonstrate that traffic signal optimization and intersection design improvements can significantly enhance traffic efficiency and reduce environmental impacts at congested urban intersections in Mansoura.

## Introduction

The most critical problems that roads in almost every large city face are traffic congestion and peak-hour environmental pollution, which impair people’s ability to move around and carry out regular tasks. Traffic congestion results from an annual increase in vehicle volume that is disproportionate to available road capacity, population growth, and the concentration of major commercial and residential structures ^1^.

Egypt, with several densely populated metropolitan areas—most notably Greater Cairo—experiences severe traffic congestion. Greater Cairo, with a population exceeding 22 million, is among the most congested metropolitan regions worldwide, with average traffic speeds of 16–20 km/h. During peak hours, commuting can take 2–3 h, and in some districts, traveling only a few kilometers can take over an hour. Traffic congestion is caused by flaws in Egypt’s public transportation system, such as delays, crowding, and poor maintenance, as well as urban planning problems, such as the concentration of significant commercial and residential developments in densely populated metropolitan areas, in addition to population growth and the yearly increase in vehicle volumes. When combined, these factors increase travel times and exacerbate urban traffic congestion ^2^.

As of 2023, the number of registered vehicles in Egypt had surged to 10 million, with a steady annual growth rate of 5–7%, according to the Central Agency for Public Mobilization and Statistics (CAPMAS). This expansion is attributed to growth in the private automobile sector, as more residents prefer personal vehicles to public transit. Egypt’s public transportation system relies on public buses and private microbuses, but it suffers from delays, congestion, and inadequate maintenance ^3^. The Egyptian Transport Authority operates approximately 3,300 buses but frequently fails to meet growing demand. Egypt is among the world’s most polluted cities, primarily due to traffic congestion. The town consistently exceeds safe PMx thresholds, where PMx denotes the total mass of particulate matter emissions estimated by the SUMO simulation, encompassing all particle sizes modeled by the emission module. However, it does not explicitly distinguish between PM10 and PM2.5, as SUMO’s default HBEFA-based emission models typically report aggregated particulate matter as a single value (PMx) unless specifically parameterized otherwise. Compromising air quality and public health ^4^, Egypt ranks among the leading cities worldwide for air pollution, primarily attributable to traffic congestion. The nation consistently exceeds allowable particle matter (PM) limits, significantly jeopardizing air quality and public health. The most recent World Health Organization (WHO) standards (2021) stipulate annual limits of 5 µg/m^3^ for PM2.5 and 15 µg/m^3^ for PM10. In numerous Egyptian urban areas, PM10 concentrations frequently exceed 100 µg/m^3^, whereas PM2.5 levels range from 35 to 60 µg/m^3^, placing Egypt well above international health-based air quality benchmarks. This underscores the pressing necessity for sustainable traffic management strategies to alleviate pollution and enhance urban livability^5–7^.

The Egyptian government is investing in infrastructure initiatives to alleviate traffic congestion, including expanding the Egyptian Metro and improving road networks. The New Administrative Capital project aims to decentralize traffic and relocate government operations to a new city, while deploying intelligent traffic systems and public transportation alternatives to mitigate congestion. Intelligent Transportation Systems (ITS) are potential remedies for these problems ^8^. These techniques reduce traffic light wait times and improve driving quality by optimizing intersection traffic flow and scheduling, thereby reducing travel times, traffic congestion, and pollutant emissions ^9^. Intelligent Transportation Systems (ITS) used in traffic refinement can incorporate Genetic Algorithms (GA)^10^, Particle Swarm Optimization (PSO)^11^, Machine Learning^12^, Reinforcement Learning ^13,14^, Fuzzy Logic and Neural Networks^15,16^.

In recent years, academic researchers have used simulation to evaluate the effectiveness of traffic networks, thereby saving time and reducing costs without modifying the infrastructure. In this context, "Simulation of Urban Mobility" (SUMO) is an open-source, microscopic, multi-modal traffic simulation software created by the German Aerospace Center (DLR). It meticulously models individual vehicles, pedestrians, traffic flow, traffic signals, public transportation, and other traffic entities. The software enables the modeling of customized routes, road configurations, and vehicle behaviors. SUMO is used for traffic planning, management, and research, facilitating activities such as traffic-light optimization, route planning, and emissions and energy-consumption modeling. SUMO uses structured geographic data (e.g., from OSM) to construct road networks (.net.xml) and traffic demand (.rou.xml). In sumo-gui, tools such as Netconvert facilitate OSM data processing, while Google Earth imagery aids visualization and validation^17^.

**Fig. 1:**
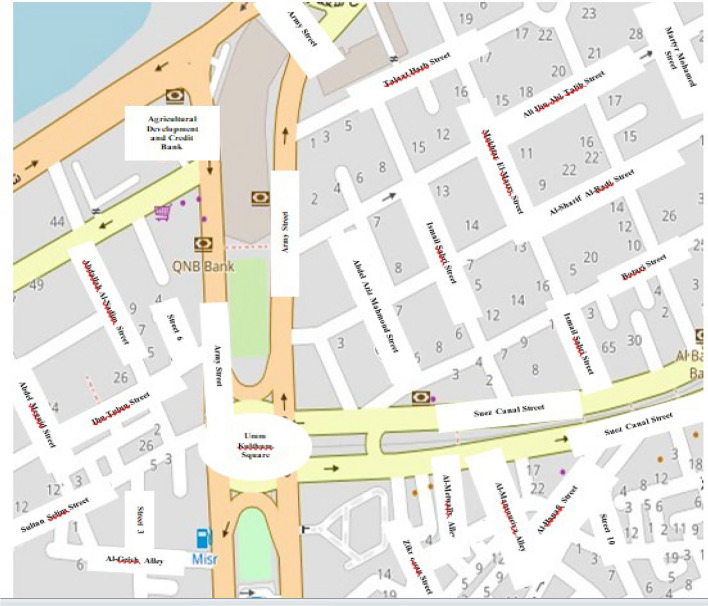
Representation of Egypt, Mansoura, and Umm Kulthum Square using QGIS (3.28) with OpenStreetMap (OSM) data^35,36^.

The OpenStreetMap ^18^ digital network is integrated to receive traffic information via data requests and updates. OpenStreetMap is a free, user-created digital map, as shown in Fig. 1, which presents statistics from Egypt, where Arabic is the official and most widely spoken language. The original information, including signage, labels, and text, is presented in Arabic and accurately reflects real-world conditions pertinent to our study’s environment. So, we translated street labels. Vehicle location is estimated from positional data, such as the longitude and latitude for each path. Next, a vehicle’s location data will be continuously updated within the Kalman filter to remove spurious data ^19^. The primary components needed to create the traffic simulation are (i) network data made up of roads and intersections, also known as edges and junctions; (ii) traffic infrastructure, which includes logic and traffic light elements; (iii) vehicle type, which includes a description of the characteristics of the vehicle (e.g., gas/diesel, passenger/bus); and (iv) vehicle traces, which are the routes, trips, and flows that the vehicles will take during the simulation.

Controlling the timing of traffic signals at junctions is a potential strategy to mitigate the effects of traffic congestion; consequently, it affects the duration of automobile travel and the environment by increasing emissions. Two forms of pollution from car exhaust are noise and environmental pollution. Vehicle emissions, including carbon monoxide (CO), carbon dioxide (CO2), nitrogen oxides (NOx), hydrocarbons (HC), and particulate matter (PMx), constitute a form of environmental pollution. There are no preventative measures or remedies for such air pollution in Egypt.

This study aims to assess the efficiency of urban traffic control in Umm Kulthum Square, a central commercial district in Mansoura, Dakhalia Governorate, Egypt. Umm Kulthum Square is home to several key institutions, including the Mansoura government office, Dakahlia Governorate Building, Dakahlia Governorate Museum, the Martyrs of Dakahlia Governorate, Grand Hospital Mansoura, Nasr Mosque, Mansoura Community Garden, the Old Security Directorate Mansoura, Mansoura Medical Center, and Ahmed Zewail International Preparatory School for Girls, The everyday operations of these institutions, in conjunction with those of commercial businesses and public services, result in high levels of vehicular and pedestrian traffic, particularly during peak hours. This is a substantial contributor to local congestion.

### The main contributions of this paper are:

1. Proposing an effective urban traffic control model for Umm Kulthum Square.
2. Simulating traffic signals and junctions using Simulation of Urban Mobility (SUMO) software.
3. Creating and modifying two scenarios for the study area, scenario 1 represents the existing traffic conditions, with the road network geometry and lane count adjusted to real-world dimensions derived from satellite imagery. Scenario 2 illustrates a modified traffic management scenario, in which lane connections, turning movements, and traffic signal configurations (including location, phase duration, and signal states) were optimized to improve traffic flow.
4. Calculating the average absolute value of emissions (CO, CO_2_, HC, PMx, NOx, and fuel) consumed by vehicles during their journeys.
5. Evaluating the efficiency of the proposed model by comparing two scenarios and estimating parameters such as average departure delay, arrival time, waiting time, duration, time loss, queuing time, queuing length, and experimental queuing length for all vehicles.

We emphasize that the strength and novelty of our study lie in its application to the Egyptian city of Mansoura, a relatively rare focus in traffic and environmental research. Specifically, our work utilizes the SUMO simulation software to assess pollution levels in this urban context—a topic that has been scarcely studied at the local level in Egypt. This localized application provides valuable insights and fills a notable gap in the literature, highlighting the practical relevance and originality of our study.

The subsequent sections of this work are structured as follows: Section "The related work" reviews the relevant literature on road traffic optimization. Section "Proposed model" outlines the recommended simulation methodology to improve the traffic light system. Section "Experimental and Results" outlines the experimental findings. Section "Conclusion" closes the work and delineates avenues for future research.

## The related work

Urban areas in Egypt experience severe traffic congestion due to rapid population growth and rising vehicle ownership, with few real-time traffic control systems in place. Simulation software provides a cost-effective means of assessing and addressing this issue. Researchers have suggested various methods for controlling traffic signal timings at junctions to alleviate congestion, emphasizing the need for dynamic adjustments to minimize wait times. Strategies for optimizing traffic signals include genetic algorithms, particle swarm optimization, machine learning, fuzzy logic, and reinforcement learning [ [20. Following a comprehensive literature review, we were unable to identify any published research from Egypt that has used the SUMO simulation framework to optimize traffic signals in conjunction with emission estimation. To provide the essential methodological basis and highlight the novelty of the proposed work in the Egyptian context, the literature review focuses primarily on pertinent international studies. This clarification has been added to the revised manuscript ^21^.

The existing literature can be classified according to its primary objectives. A large group of studies concentrates on adaptive and intelligent traffic signal control, including works by Andrea et al., Seyit et al., and Tuo et al., who explore optimization techniques based on genetic algorithms, swarm intelligence, and machine learning, as well as Muzamil et al. and José et al., who apply fuzzy-logic-based strategies for dynamic signal timing. Reinforcement-learning-driven approaches are investigated by Luow et al., Dimitrius et al., Xiaoyi et al., and Carvalho et al., addressing distributed control, algorithmic improvements, comparative evaluation, and hierarchical decision-making, respectively. The credibility of traffic simulation is examined by Ilhan et al. through the validation of SUMO-generated traffic against real-world data. Another research direction focuses on vision-based vehicle detection to support innovative traffic systems, as demonstrated by Baena et al. for trajectory extraction and by Al-Zoghby et al. for real-time adaptive control using visual traffic information. In addition, Biramo et al. address sustainability and environmental impact analysis, assessing fuel consumption and emission reductions associated with varying levels of autonomous vehicle penetration, as shown in the Table. 1.

**Table 1:** Overview of related work.

Ref	Main Target / Objective	Methodology	Dataset / Simulation	Metrices	Key Results	Pros	Cons / Limitations
Andrea et al	Enhanced comprehension of traffic signal scheduling	Cellular Genetic Algorithms (CGAs), in-depth solution analysis	Synthetic traffic scenarios	Convergence velocity, solution variability, transit duration	CGAs outperform conventional GAs in both convergence and diversity	Offers a profound understanding of the solution space and is adaptable to complex intersections	Characterized by high computational demands, predominantly reliant on simulations, and may lack applicability to real-time scenarios
Seyit et al	Real-time adaptive traffic signal control	Swarm Optimization: PSO, ACO	SUMO traffic simulator	Average waiting time, queue length, travel time	Substantial decrease in wait times and queues	Capable of real-time operation; straightforward implementation; adaptable	Vulnerable to algorithmic settings; efficacy may diminish under inconsistent traffic conditions
Tuo et al	Improve GA-based traffic control using ML	Boosted Genetic Algorithm guided by Machine Learning	SUMO simulation	Travel time, delay, throughput	Hybrid GA-ML reduces transit time and delays, thereby accelerating convergence	Combines ML guidance with GA to increase convergence and efficiency	Significant complexity; necessitates training data; extended configuration duration
Muzamil et al	Compare fuzzy logic strategies for traffic signal timing	Fuzzy logic controllers with state inputs	SUMO simulation	Throughput, delay, stops	State-based fuzzy controllers improve traffic throughput	Flexible and interpretable; can model nonlinear traffic patterns	Manual rule creation involves specialized knowledge, and performance is susceptible to adjustment
José et al	Adaptive traffic control using fuzzy logic + classical formulas	Fuzzy logic combined with Webster and Modified Webster formulas	SUMO traffic simulator	Delay, stops, and queue length	decreased stops and delays in comparison to fixed-time control	Combines traditional and sophisticated methods; adaptable	Depends on precise traffic estimation; simulation-based
Luow et al	Distributed cooperative intersection control	Multi-agent Reinforcement Learning (MARL)	SUMO simulation	Delay, travel time, throughput	Cooperation increases intersection efficiency and lessens traffic	Decentralized; scalable to multiple intersections	Training stability issues; high complexity
Dimitrius et al	RL-based traffic signal optimization with dual agents	Dual-agent Double Deep Q-Network (DDQN)	SUMO simulation	Travel time, waiting time, convergence rate	Faster convergence and reduced delays vs standard DQN	Addresses overestimation; efficient learning	Requires a large training dataset; computationally intensive
Xiaoyi et al	Compare reinforcement learning agents for traffic signal optimization	Comparative study of RL agents (DQN, DDQN, etc.)	SUMO simulation	Travel time, delay, throughput	Identified the strengths and weaknesses of different RL agents	Provides benchmarking insights; practical guidance	Results scenario-dependent; may not generalize universally
Carvalho et al	Hierarchical traffic signal control using RL	Hierarchical Reinforcement Learning (Options Framework)	SUMO simulation	Travel time, waiting time, and learning efficiency	Improved learning efficiency; better long-term decision-making	Scalable; handles complex decision sequences	Increased algorithmic complexity; longer training required
Ilhan et al	Validate the accuracy of SUMO traffic flow generation	Statistical comparison of simulated vs. real traffic	Real-world traffic data; SUMO simulation	Traffic volume, speed, and flow patterns	SUMO closely replicates real traffic behavior	Validates SUMO for research; important for simulation-based studies	Not a control method; limited to flow validation
González et al	Estimate road pollution from vehicle movements using video surveillance	Deep Convolutional Neural Networks for vehicle detection and tracking; trajectory-based emission estimation	Surveillance traffic videos; UNLV dataset; traffic flow analysis	Recall, vehicle trajectory accuracy, and turning movement counts (TMC)	Achieved a recall ≈ of 0.62; reliable vehicle trajectories enabling pollution estimation	Non-intrusive pollution monitoring uses existing cameras and supports environmental traffic analysis	Sensitive to lighting and weather; limited real-time performance; struggles in dense or complex scenes
Al-Zoghby et al	Real-time adaptive traffic management to reduce congestion and emissions	YOLOv11-based vehicle detection; traffic density estimation; dynamic signal optimization	Real-time camera feeds; traffic simulation for performance evaluation	mAP, F1-score, waiting time, fuel consumption, emissions	High detection accuracy (mAP 92.4%, F1 89.7%); significant reduction in waiting time and emissions	Real-time capable; high accuracy; integrates traffic efficiency with sustainability goals	Performance degrades under severe weather and very low-light conditions
Biramo et al	Evaluate the impact of automated vehicles on fuel consumption and emissions	Traffic microsimulation under different AV penetration and automation scenarios	22-km simulated test track; five AV penetration scenarios	Fuel consumption, CO₂ emissions, traffic flow indicators	25% AV penetration reduced fuel and CO₂ by up to 8.35%; higher penetration yields greater benefits	Quantifies environmental impact; beneficial for transport planning and policy analysis	Entirely simulation-based; assumes predefined driving behavior; no real-world validation

Andrea et al. ^22^ introduced synchronous and asynchronous Cellular Genetic Algorithms (CGAs) for traffic signal scheduling in the SUMO micro-simulator. Implemented in Java and tested in real-world scenarios in France and Spain, their approach demonstrated strong performance without requiring costly infrastructure upgrades or drivers installing special software.

Seyit et al. ^23^ recommended the particle swarm optimization (PSO) method to optimize real-time traffic signal control. The proposed technique uses real traffic data from Kilis, Turkey. SUMO traffic simulator is used to assess performance in real time. The success of the proposed method is quantified by comparing the results obtained with the actual traffic measurement data. Nevertheless, these systems ^22,23^ require substantial resources, such as time or processing power, to determine the most efficient signaling technique.

Tuo et al. ^24^ proposed a traffic signal optimization strategy that combines machine learning with genetic algorithms to minimize the impact of accidents on total travel time (TTT). Their approach achieved an estimated 50% reduction in TTT compared to the original genetic algorithm ^22^. Although linear regression is widely used in traffic control, its application is challenging because it has few parameters and is a parametric model. It is not robust, has poor accuracy, and performs poorly on nonlinear traffic controllers.

Muzamil et al. ^25^ developed a fuzzy logic-based adaptive traffic signal control system using Webster’s formula and its modified version. Evaluation using SUMO showed that the system improved average speed, waiting time, and trip duration at a four-way intersection, outperforming traditional methods. With the techniques mentioned above ^26,27^, precise reasoning is typically impossible because implementing fuzzy logic requires significant human expertise and frequent rule updates. Moreover, there is no assurance of stability or optimality, and the performance depends on the heuristic parameters and rules.

José et al. ^26^ proposed a distributed traffic control strategy based on reinforcement learning (RL) that leverages cooperation among intersections. The system uses traffic predictions to inform RL controllers that manage signal timings. SUMO simulations showed that this method outperforms traditional approaches in reducing waiting times and improving overall performance metrics.

Luow et al. ^27^ proposed optimizing the queue length at intersections using Q-learning and policy optimization, two machine-learning algorithms employed. The agents can view the traffic light’s current state and the number of vehicles. This method disregards environmental aspects that should be considered for this work.

Dimitrius et al. ^28^ proposed an options framework with hierarchical reinforcement learning (HRL) to optimize traffic-light control. Their method uses sub-policies to improve traffic flow and reduce waiting times. While the results show improvements over fixed-time models, the approach was tested only in simulations on a simplified intersection. Therefore, further research is needed before real-world application.

Xiaoyi et al. ^29^ used a traffic simulation model of China to address recurring traffic jams in eight regions of Wuhan. The simulation provided real-time, precise measurements of traffic conditions and estimates derived from prior information, thereby aiding traffic signal management and road planning.

Carvalho et al. ^30^ introduced "Lightweight PVIDNet," a deep learning model for real-time identification of priority vehicles to enhance traffic signal systems and emergency response under the Brazilian Traffic Code. Trained on 5,250 Brazilian photos with TensorFlow/Keras, it improves YOLOv3 with DenseNet and Soft-Root-Sign activation, achieving classification accuracy of 0.95–0.97 and processing at 34.25 FPS. This model reduces waiting time by 50% and travel times for priority vehicles by 45%, while maintaining normal traffic flow. Its lightweight design enhances compatibility with edge devices and outperforms conventional models in accuracy and speed, although further validation in real-world scenarios is needed to optimize it.

Ilhan et al. ^31^ proposed a fuzzy logic controller (FLC) for traffic light control at a four-way intersection, using queue length and vehicle location as inputs. Simulated in SUMO, the FLC outperformed fixed-time signals in reducing waiting time and queue length. However, the method struggles with complex junctions and multiple intersections.

González et al. ^32^ introduced a methodology that integrates computer vision with the Simulation of Urban Mobility (SUMO) to improve traffic flow modeling and signal performance evaluation. Their approach employs the YOLOv5 object detection model to track vehicle and pedestrian movements captured by traffic cameras. This methodology estimates turning movement counts (TMCs), which are then fed into SUMO simulations to analyze intersection performance. While achieving a recall of 0.62, the YOLOv5 tracking system has limitations, including sensitivity to environmental factors, constrained real-time processing, and challenges in complex scenarios.

Al-Zoghby et al. ^33^ introduced a Smart Traffic Management System (STMS) that employs a YOLOv11-based deep learning model for real-time vehicle identification and traffic-flow enhancement in smart cities. This system uses computer vision and AI to analyze real-time traffic, classify vehicles, and adaptively control traffic signals, thereby reducing journey times, fuel consumption, and emissions. Achieving a mean average precision of 92.4% for vehicle detection, with high accuracy for buses (0.92) and vehicles (0.91), the STMS also demonstrated an F1 score of 89.7% in varying conditions. It effectively decreased congestion and wait times, thereby promoting urban mobility and sustainability. However, its performance can be adversely affected by severe weather or low-light conditions.

Biramo et al. ^34^ investigated the effects of autonomous vehicles (AVs) on emissions and fuel consumption via a microsimulation model on a 22-km track, evaluating five scenarios with varying AV penetration rates and automation levels during peak and non-peak traffic. The study highlights the ecological advantages of AVs, emphasizing the importance of driver behavior and traffic conditions in reducing emissions. Findings indicate that a 25% AV penetration with limited automation can reduce fuel use and CO₂ by 8.35%, with higher AV penetration and automation yielding greater emission reductions, particularly in peak traffic.

## Proposed model

**Fig. 2:**
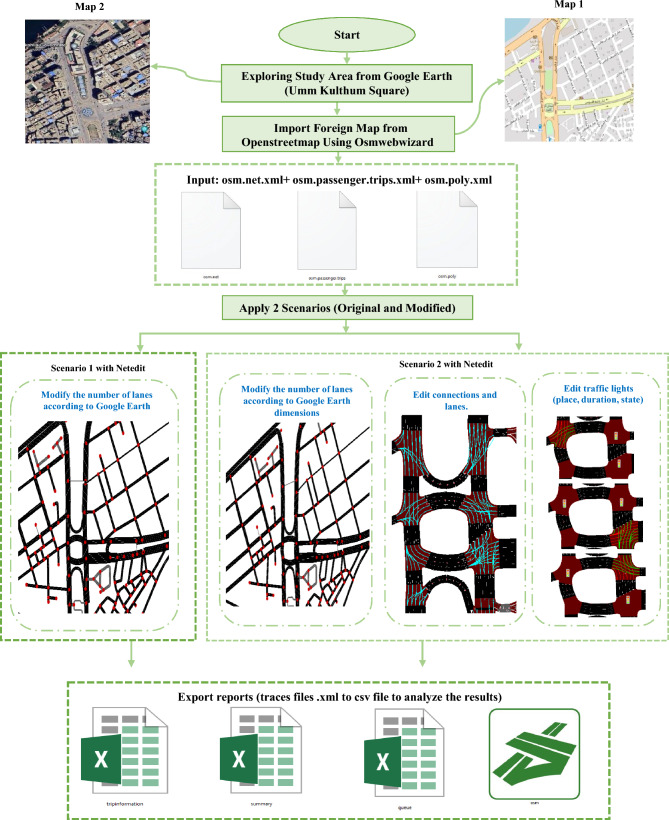
The Proposed Framework for Umm Kulthum Square, Mansoura city, Dakahlia Governorate, Egypt 2024, (**a**) Map1: from QGIS (3.28) with OpenStreetMap (OSM Data ^35,36^, (**b**) Map 2: from Google Earth at Coordinates 31°02′58"N 31°23′43"E.

The proposed system uses the SUMO (Simulation of Urban Mobility) traffic simulator to model and analyze traffic flow in Umm Kulthum Square, a central commercial and administrative hub in Mansoura, Dakahlia Governorate, Egypt. The system integrates high-resolution satellite imagery from Google Earth and geospatial data from OpenStreetMap (OSM) to create a detailed digital representation of the study area, which includes traffic intersections, roads, and traffic lights. The system evaluates traffic performance under two scenarios—original (Scenario 1) and modified (Scenario 2)—by adjusting lane configurations and traffic light settings to resolve conflicts and reduce vehicle waiting times. Settings include location, cycle duration, and phase states to prioritize main flows and minimize delays. Moreover, it generates detailed reports of comprehensive performance metrics, including Queue Metrics, Trip Information, and Summary Metrics, and also produces emission reports using the HBEFA model. SUMO calculates emissions (CO₂, NOx, PMx, HC, CO, and fuel in mg) and noise levels based on vehicle types, traffic conditions (e.g., urban stop-and-go), and driving profiles as shown in Fig. 2.

## Exploring the study area

Acquiring an image of the study area is crucial, as it will help delineate our network and identify key components in SUMO. We used Google Earth to collect data and map our study area using satellite imagery. This imagery can be analyzed to extract coordinates and visually assess road infrastructure, thereby aiding the verification of OpenStreetMap (OSM) data, for example, by examining road alignments or detecting missing features. Furthermore, it improves display within SUMO’s graphical interface (SUMO-GUI). We used the ‘Ruler’ tool in Google Earth to measure dimensions precisely, using meters as the unit. Accurate measurements are essential for our research. Google Earth offers high-resolution imagery that provides superior spatial context; however, the quality may vary by region and date. It is worth noting that Google Earth does not provide organized geographic data comparable to OSM and cannot directly produce precise features, such as highway classifications, maximum speed limits, or lane counts. SUMO will process all the data and simulate our models using these measures. The width is 401 m, and the height is 77 m.

**Fig. 3:**
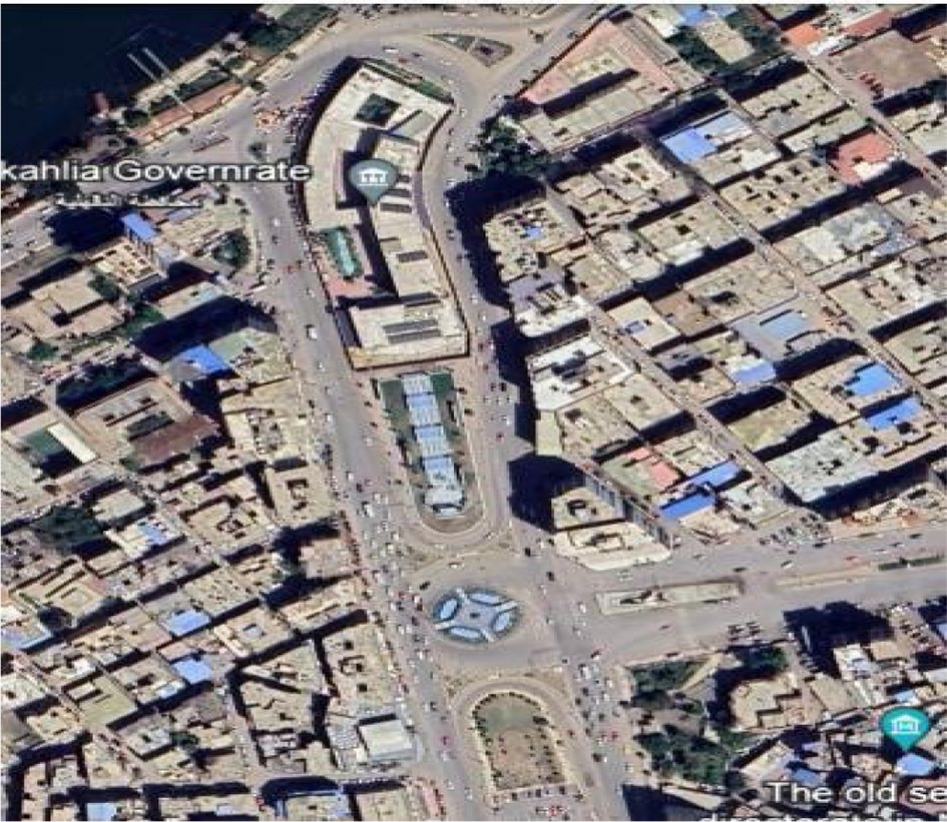
Umm Kulthum Square, Mansoura city, Dakahlia Governorate, Egypt 2024 from Google Earth at Coordinates 31°02′58"N 31°23′43"E.

Let us tour the selected area by using Google Earth to look for Egypt, Mansoura, and Umm Kulthum Square is located at coordinates (25°25′05"S 49°15′43"W ) ^35^, as shown in Fig. 3, which is one of the primary squares in Mansoura, Dakhalia Governorate, Egypt, also a significant commercial district that is home to several offices, hospitals, and shopping malls; it is interesting for our research because there we can find traffic intersections, traffic lights and simulated it with SUMO.

In our paper, we will visit the area and illustrate the most important aspects; hence, we segment the area into eight sectors. These sectors were not arbitrarily chosen; instead, they were selected based on their functional, geometric, and traffic-control diversity surrounding Umm Kulthum Square. These sectors collectively represent typical traffic patterns, road geometries, and intersection configurations within this key urban node. Considering that the Local roads often have narrower lanes than Arterial roads, ranging from 2.5 to 3.0 m. Shoulder widths on Local roads are typically 2.0–3.0 m. Sidewalks may vary in width but are typically 1.5–2.5 m. Intersection dimensions vary, but turning lanes may have widths of 3.0–4.5 m ^36^, as shown in Table 2.

**Table 2:**
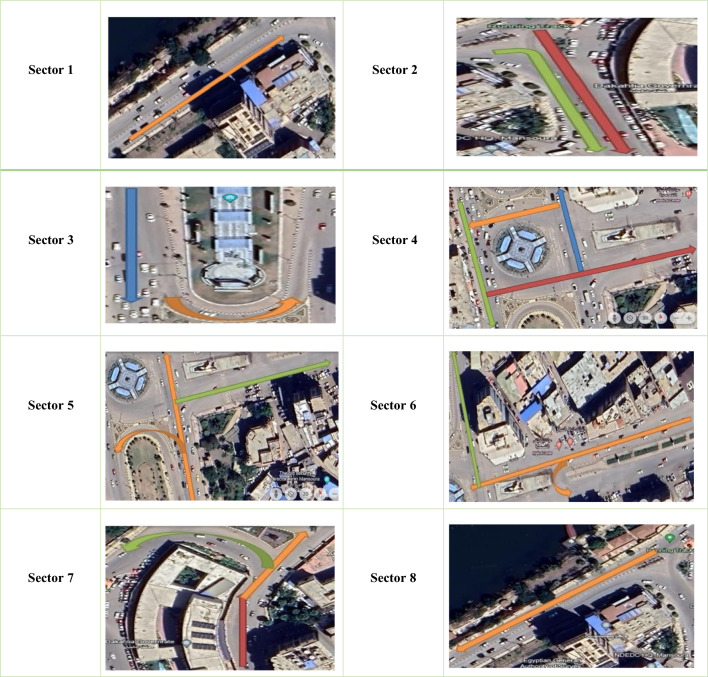
Umm Kulthum Square Sectors, Mansoura City, Dakahlia Governorate, Egypt 2024 from Google Earth at Coordinates 31°02′58"N 31°23′43"E.

A. (Sectors 1–3) cover the primary administrative and commercial frontages, including areas with dynamic lane configurations, government buildings, and varied road widths.
  Sector 1
    Here, we have two stretches as in the orange path; in the first stretch, we have three lanes with a width of 8.21 m; when moving some meters in the second stretch, we notice the road widening to four lanes with a width of 13.15 m, and we are now in front of the Mansoura government office.
  Sector 2
    After moving a few meters, we are now in front of the Dakahlia Governorate building, an essential administrative structure. Here, vehicles approach from two directions: one four-lane, 12.51 m wide (green path), and the other two-lane, 6.84 m wide (red path). The road then widens to six lanes, each 22.37 m wide.
  Sector 3
    Here, we have five lanes 20.22 m wide in front of the Dakahlia Governorate Museum; moving some meters, we have five lanes 21.83 m wide in front of the memorial to the martyrs of Dakahlia Governorate, as in the blue path and later U-Turn, found two lanes with 5.85 m wide, as in the orange path, and a traffic light.
B. Sectors 4 and 5 involve complex intersections with multiple traffic lights and lane merging and splitting scenarios, which are critical for traffic flow modeling.
  Sector 4
    We find a traffic intersection with three traffic lights through 4 lanes (14.3 m approximately wide in front of Grand Hospital Mansoura and Nasr Mosque, as in the green path, 14.6 m approximately wide as in the red path, and 13.74 m approximately wide as in the orange path), and six lanes with 20.44 m approximately wide as in the blue path. Later, we will present an illustrative example of identifying possible movements and locating traffic lights, as shown in Tables 3 and 4
  Sector 5
    Let us change the direction. We are now following from South to North. We have six lanes, each 21.55 m wide, as in the orange path that contains a U-turn in front of the Mansoura community garden, and two lanes approximately 7.09 m wide, as in the orange path. Then the road narrows to five lanes, each 18.76 m wide. At the end, there is a traffic light; hence, we have a clear direction with seven lanes, approximately 27.3 m wide, as if on a green path.
C. Sectors 6 and 7 illustrate the variability in directionality, lane usage, and school-related traffic, which influence peak-hour flows and road behavior.
  Sector 6
    As in sector 5, in the opposite direction, we have three lanes: one extra lane for left-turning vehicles at 14.76 m, and the road then narrows to three lanes at 10.82 m in front of Mansoura Medical Center, as shown in the orange path. At the end, there is a traffic light; hence, we have the right direction with four lanes 12.90 m wide, as if they were on a green path.
  Sector 7
    As in sectors 3 and 5, we have five lanes 17.82 m wide (as in the red path) and one additional lane for left-turning traffic. At Ahmed Zewail International Preparatory School for Girls, the road branches into two directions: one with three lanes, 10.52 m wide, as in the Orange Path, and the other with a U-turn and four lanes, 13.81 m wide, heading to sector eight, as in the Green Path.
  Sector 8
    Completes the loop, capturing the return flow and summarizing the typical lane configurations in the opposite direction to Sector 1. It has three lanes, 10.91 m wide, as in the orange path.
    These sectors were thus selected to encompass the entire perimeter and radial roads of Umm Kulthum Square, to include a mix of arterial and local roads, intersections, U-turns, and traffic control mechanisms, and to provide a representative dataset for modeling in SUMO, enabling the simulation of meaningful traffic behavior.

**Table 3:**
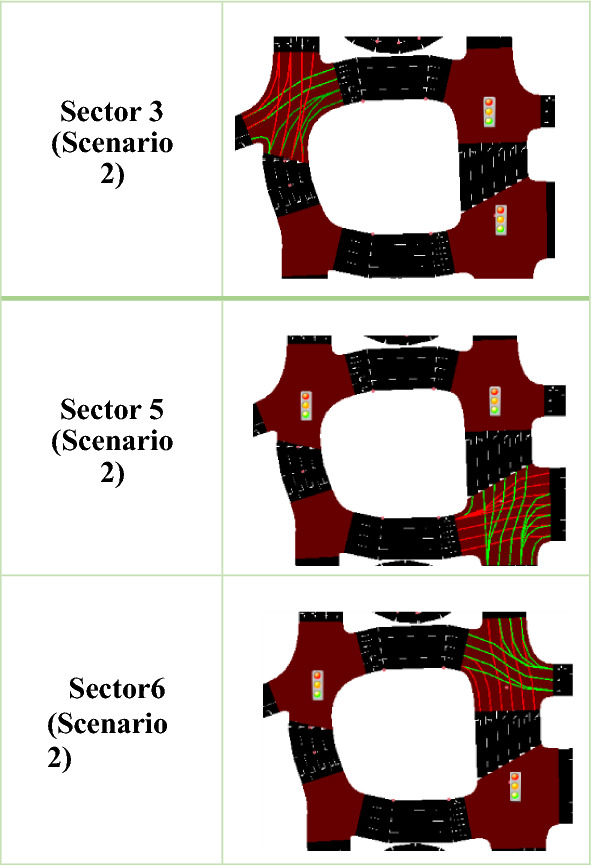
Traffic Lights Editing in Scenario 2.

**Table 4:** Criteria for Adjusting Traffic Signals in Scenario 2.

	Paths	Duration (second)	State
Sector 3	0	33	rrrrrrGGggggGG
	1	6	Rrrrrryyggggyy
	2	6	rrrrrrGGGGGGrr
	3	6	Rrrrrryyyyyyrr
	4	33	GGgGGGrrrrrrrr
	5	6	yyyyyyrrrrrrrr
Sector 5	0	50	GGGGGGGGGGrrrrrrrG
	1	6	yyyyyyyyyyrrrrrrry
	2	30	grrrrrrrrrGGGGGGGG
	3	6	rrrrrrrrrryyyyyyyy
Sector 6	0	39	GGGGGGGrrrrrr
	1	6	yyyyyyyrrrrrr
	2	60	rrrrrrrGGGGGG
	3	6	rrrrrrryyyyyy

### Import foreign map for OpenStreetMap

The avenue’s topological data was successfully integrated into the urban traffic simulation environment via OpenStreetMap (OSM) ^37^, a volunteer-managed open geospatial database. Using native SUMO scripts, the application successfully exported a digital map that encompasses diverse elements, including road geometry (nodes and ways), road classifications (e.g., highways, residential areas), lane specifications, speed restrictions, and traffic signals.

We generated our simulation by importing our research area into SUMO using OSM and obtained the required data by executing the Osmwebwizard function within SUMO. The OSM data was obtained directly from the OSM website by selecting a rectangular region. After obtaining the OSM data, SUMO transformed it into a format suitable for traffic simulation ^38–40^. The tool used for this conversion is netconvert, which converts OSM’s XML files to SUMO’s network file format (.net.xml). During scenario design, SUMO was automatically initialized with the map, and the simulation commenced. As a result, we identified several simulation files within the SUMO directory on our computer, including osm.net.xml, osm.passenger.trips.xml, osm.poly.xml, osm.view.xml, and osm. sumocfg, and osm. polycfg, the osm. The sumocfg file functions as the primary configuration file, integrating three essential files as inputs ^41^:

- osm.net.xml: encompasses critical data regarding edges, junctions, and connections inside the network.
- osm.passenger.trips.xml: delineates the route and trip specifics for each vehicle.
- osm.poly.xml: delineates the dimensions and configurations of edifices and supplementary structures on the map.

This method is openly available and collaboratively enhanced, featuring extensive tagging for roads and infrastructure. Although suitable for limited spaces, data density often restricts downloads to areas of less than one square kilometer in highly populated areas. Furthermore, it may not accurately reflect recent modifications to road networks or changes in traffic laws. To resolve this issue, we performed a manual validation by examining the network in SUMO-GUI and superimposing it with Google Earth imagery to detect missing roads, erroneous geometry, or misaligned traffic signals.

### Apply two scenarios (Original and modified)

After exploring the study area, Umm Kulthum Square, using satellite imagery from Google Earth to accurately identify road dimensions and intersection layouts, these visual insights are combined with data obtained from OpenStreetMap (OSM) to generate the core SUMO input files, which include the network file (osm.net.xml), vehicle trip file (osm.passenger.trips.xml), and polygon file (osm.poly.xml) for background elements and visual context.

Using these inputs, two distinct simulation scenarios are created: an original scenario and a modified one. In Scenario 1, the network is edited in SUMO’s graphical tool, NetEdit, to adjust lane counts based on real-world measurements from Google Earth. Scenario 2 further modifies the network by editing lane connections and traffic signal configurations—including their location, phase durations, and state sequences—to reflect optimized or hypothetical changes in traffic control.

**Fig. 4:**
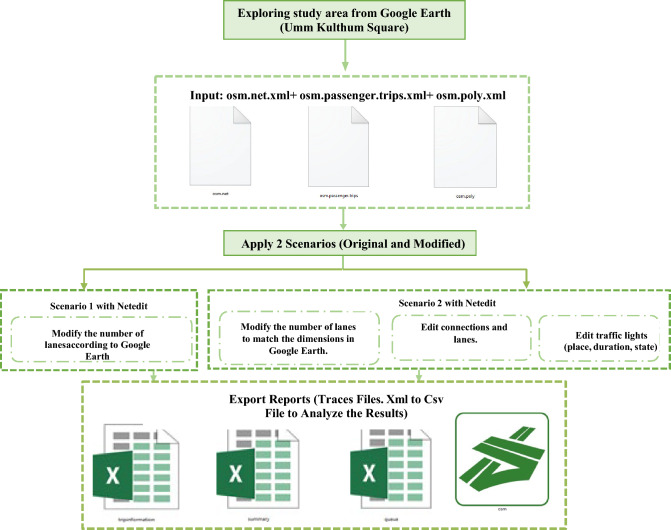
Proposed Two Scenarios (Scenario1, Scenario2).

Finally, both scenarios are executed in SUMO, and the resulting trace files (in XML format) are exported and converted to CSV for post-simulation analysis. These output files are used to extract quantitative metrics such as travel time, congestion, and emissions, enabling a comparative evaluation of the original and modified traffic scenarios, as shown in Fig. 4.

####  Scenario 1 (Original)

The specific part of the OpenStreetMap was imported, and by deleting unimportant elements and backgrounds, and with modifications to the network in several lanes according to Google Earth dimensions, we extracted the output files (Floating Car Data (FCD) trace file, Raw Vehicle Positions Dump, Emission Output, Full Output) as output trace files.

####  Scenario 2 (Modified)

Also, after importing the map from OpenStreetMap and deleting unimportant elements and backgrounds, we modified the network in terms of the (number of lanes according to Google Earth dimensions, connections, lane directions, and traffic lights (place, duration, state), also extracted the files (Floating Car Data (FCD) trace file, Raw vehicle positions dump, Emission Output, Full Output) as output traces files, then compared to the results of the Scenario 1.

#####  Creating network and lane modification

Let us build a SUMO network for our Study Area using NetEdit ^42^. It is challenging to construct a SUMO network that represents the real world when a reference image is unavailable, as shown in Fig. 3. To help us, let us use the pre-modeled study-area image; it will make it easier to locate junctions and edges and to learn essential attributes. Using Google Earth and the width parameter for each road sector, we adjust the number of lanes based on the dimensions.

#####  Editing connections

**Fig. 5:**
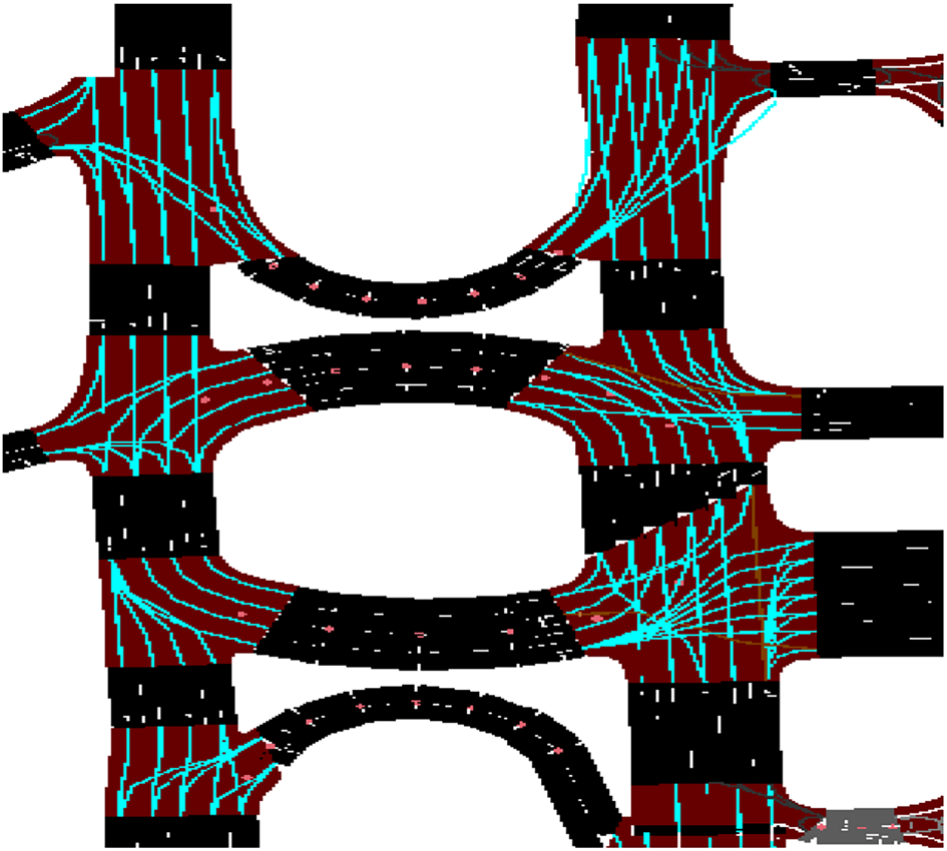
Connections Editing in Scenario 2.

In SUMO, editing connections is essential for reducing vehicle conflicts at intersections and improving the realism of traffic flow. Using the NetEdit tool, we can manually adjust how lanes are connected at junctions—removing incorrect links, assigning proper turn movements, and ensuring dedicated lanes function correctly. These adjustments help prevent overlapping paths, minimize conflicts, and support accurate simulations ^43^; therefore, we must correct them, as shown in Fig. 5.

#####  Editing traffic lights

We encounter numerous traffic conflicts, and many vehicles are stopped, waiting for an opportunity to move forward. A possible solution is to edit the timing of traffic lights in our network. To do this, in NetEdit, select 'Traffic Light Tools’ and create a TLS. You can learn more about TLS from ^42^. By default, all TLSs are generated with a fixed cycle; therefore, we adjust the TLS settings and optimize our flows to avoid conflicts. TLS includes the place, duration, and state (phase), and we finally save the network, as shown in Table 3.

Here, we use three static traffic lights in sectors 3, 5, and 6, respectively. Sector 3 has six paths, so we adjusted the duration and state to reduce waiting time. We also applied the same changes to Sectors 5 and 6, each of which has four paths. The state is defined as the traffic-light phase at an intersection that regulates multiple incoming lanes or edges. Each character represents a single connection (e.g., a turn from one route to another). The total character count corresponds to the number of regulated connections at the junction, for example, (GGG) when segmenting the string as illustrated in Table 4:

- rrrrrr: The first six lanes/connections have red lights.
- GG: The next two lanes get priority green (e.g., straight-through lanes).
- gggg: Next, forget non-priority green (e.g., minor turns or non-conflicting flows).
- GG: The last two get priority green, possibly another main flow.

###  Export reports (trace files)

The default vehicle-following model in Eclipse SUMO is an adaptation of the Krauss model, initially developed by Stefan Krauss in 1997 ^44^. This model is primarily built upon the concept of "safe speed," which is computed as follows:

1 $$V_{safe} = V_{safe} \left( t \right) + \frac{{K_{n} \left( t \right) - V_{e} \left( t \right)T}}{{2\frac{{V_{m} \left( t \right) + V_{e} \left( t \right)}}{2b} + T}}$$

(*Ve* (*t*), *Kn*(*t*), *Vm* (*t*), *b*, *T*) Eq. **1** describes the following variables: The velocity of the leading vehicle at time t, the distance between the leading and following vehicles, the velocity of the following vehicle at time t, the maximum deceleration, and the driver’s reaction time. The safety of the cars is guaranteed by Eq. 1. Nevertheless, the safe speed may exceed the designated speed limit or the vehicle’s maximum attainable speed. Consequently, the desired speed term is formulated and.

Can be computed in the following manner ^45^:

2 $${\text{Vdesired }} = {\mathrm{Mim}}({\mathrm{Vm}}({\mathrm{t}}) + a{\mathrm{t}},{\mathrm{Vsafe}},{\mathrm{V}}\lim {\mathrm{it}}$$

Equ.2, where (, t, *Vl*i*m*i ) specifies respectively the acceleration, time, and speed limit, where the desired speed is the lowest value among these limitations. To provide a more realistic driver model, an imperfection parameter is introduced. However, this parameter is sampled randomly for each vehicle at each time step, allowing variability in spacing. Therefore, the.

The velocity of the following vehicle is determined as ^46^:

3 $${\mathrm{V}}_{{{\mathrm{t}} + {\Delta t}}}^{{\mathrm{m}}} = {\text{Max }}\left( {0,{\mathrm{V}}_{desired} - \in .a} \right)$$

Equ.3, where ∈ , δ denotes the noise amplitude and a random number, enables the identification of vehicles with different velocities.

SUMO supports various traffic management methods and presents them visually, and it supports multiple emission models, including HBEFA and PHEM ^34^. SUMO conducts detailed microscopic traffic simulations, monitoring each vehicle’s speed, acceleration, and position at every time step and documenting driving patterns as input to emission models.

We apply Handbook Emission Factors for Road Transport; HBEFA supplies emission factors categorized by vehicle types, traffic conditions, and driving patterns (e.g., stop-and-go, free-flow). SUMO maps a vehicle’s movement to HBEFA traffic scenarios (e.g., "urban stop & go," "free-flow"). The necessary input data includes vehicle type (e.g., passenger car, diesel truck), engine type, Euro standard (e.g., Euro 4; Euro 6), traffic context (urban/rural/motorway), and driving profile (derived from SUMO), while the output consists of pollutants such as CO₂, NOx, PMx, HC, and CO measured in g/km or g/sec ^44^. To determine emissions, we utilize the HBEFA-based emission model of Eclipse SUMO. The power demand of the vehicle in Eclipse SUMO (B) is determined using the following method:

4 $${\mathrm{B}} = {\mathrm{C}}0 + {\mathrm{C}}1{\mathrm{V}}a + {\mathrm{C}}2{\text{ V}}a^{2} + {\mathrm{C}}3{\mathrm{V}} + {\mathrm{C}}4{\mathrm{V}}^{2} + {\mathrm{C}}5{\mathrm{V}}^{3}$$

Equ. 4 represents a polynomial function with coefficients C0, C1, C2, C3, C4, and C5. V and Va denote the vehicle’s speed (which may pertain to several types of speed, including instantaneous speed, average speed, or particular driving conditions), and B denotes the output power. The coefficients of Cn in Eq. 4 are determined based on the vehicle and engine types. The emission factors used to predict power demand are sourced from the HBEFA database. The model was constructed by obtaining data from HBEFA and adapting them to a continuous function, derived by modifying the function representing the power required by the vehicle engine to overcome driving resistance ^47,48^. A uniform functional form has been used across all emissions, with parameters varying by emission type and vehicle. SUMO enables the production of a wide variety of measures for exporting and analyzing output. As shown in Algorithm 1, we have many outputs like ^49^:

I. Floating Car Data (FCD) Trace File: is an XML-formatted log produced during simulation, one of the most basic vehicle data types that we can output from SUMO, which is an output file that contains information for each vehicle in the network at every time step, like:
  A. ID: The ID of the vehicle.
  B. Location (x, y): The provided regional projection determines the vehicle’s absolute X and Y coordinates, measured in meters.
  C. Angle: The vehicle’s angle ranges from 0 to 360 degrees, with zero at midnight, measured in degrees.
  D. Type: Type of vehicle (passenger, bus, taxi, bike).
  E. Speed: The vehicle’s speed is measured in meters per second.
  F. Position: Vehicle location is determined from the start of the present lane.
  G. Lane: The present lane id.
  H. Slope: The vehicle’s slope, expressed in degrees (which corresponds to the road’s slope at this location).
II. Raw vehicle positions dump (net state dump**):** which contains (every edge and lane, each vehicle position, and speeds) for each simulation step.
III. Emission Output: The significant output in SUMO illustrates the route, type, lane, and the amount of (CO_2_, CO, HC, NOx, fuel, and noise) emitted by each vehicle in the actual simulation step, like:
  A. CO_**2**_, CO, HC, NOx, PMx: The vehicle’s emissions during the real simulation step were measured in mg for carbon dioxide (CO2), carbon monoxide (CO), hydrocarbons (HC), nitrogen oxides (NOx), and particulate matter (PM), respectively.
  B. Fuel: During the real simulation step, the vehicle’s fuel was measured in mg.
  C. Noise: The vehicle’s noise during the real simulation step was measured in dB.
  D. Waiting: The duration of the car’s weight is measured in seconds.
IV. Full Output: Extract all information from the network, including emissions, position, speed, and lane; this job is time- consuming and results in a substantial file size (about GB). It contains travel time, CO, CO_2_, NOx, PMx, HC, noise, fuel, electricity, max speed, occupancy, and vehicle count, like:
  A. Travel time: The average journey time on that particular lane is measured in seconds.
  B. Max Speed: The maximum Speed vehicles can travel in a specific lane, measured in m/sec.
  C. Occupancy: The lane’s occupancy in percentage.
  D. Vehicles count: The number of vehicles occupying the lane.
  E. State: The condition of a traffic signal right now.
V. Queue Output: The information regarding each vehicle’s queuing time, queuing length, and queuing length experimental according to each time step.
  A. queueing_time: The overall waiting time of vehicles that have to wait in a queue is measured in seconds.
  B. queueing_length: the distance between the junction and the last vehicle in the queue, measured in meters.
  C. queueing_length_experimental: the distance in the queue or how long it will take for the last vehicle to go at a speed under 5 km/h.
VI. Trip Information**:** The information regarding each vehicle’s departure delay time and arrival time for each car, and other information, will be illustrated here.
  A. Depart delay (sec): the vehicle’s waiting time before it could begin its trip.
  B. Arrival (sec): when the vehicle arrives at its location.
  C. Duration (sec): the car’s time to complete the route.
  D. Waiting time (sec): the time (planned pauses excluded) during which the vehicle’s speed was less than or equal to 0.1 m/s.
  E. Time loss (sec): the time lost due to traveling slower than recommended.
  F. speed_avg (m/sec): average vehicle speed within the trip.
  G. Abs (CO, CO_**2**_, HC, PMx, NOx, fuel): absolute fuel value represents the amount of fuel consumed during the journey (CO, CO_2_, HC, PMX, NOX, fuel).
  H. Noise_avg (mg): average vehicle noise within the trip.
VII. Summary: The number of vehicles that are loaded, inserted, operational, awaiting insertion, and have arrived at their destination, as well as the time taken to complete the route, are all included in this output. The last value is normalized across all vehicles that have arrived at their destination thus far, as illustrated here.
A. Mean Waiting Time (sec): All vehicles had to wait to be inserted during this period.
B. Mean Travel Time (sec): As previously reported, the average trip time for every vehicle exited the simulation.
C. Loaded: The total number of vehicles imported from input files up to this time step.
D. Inserted: Total number of vehicles inserted to this time step.
E. Waiting: The number of vehicles awaiting insertion but could not be inserted within the specified time frame.
F. Mean speed (m/sec): The mean Speed of all the non-waiting vehicles in the network.
G. Ended: Number of vehicles that were either eliminated from the simulation or that have already arrived at their destination.
H. Arrived: The number of vehicles that have already arrived at their destination.

**Algorithm 1:**
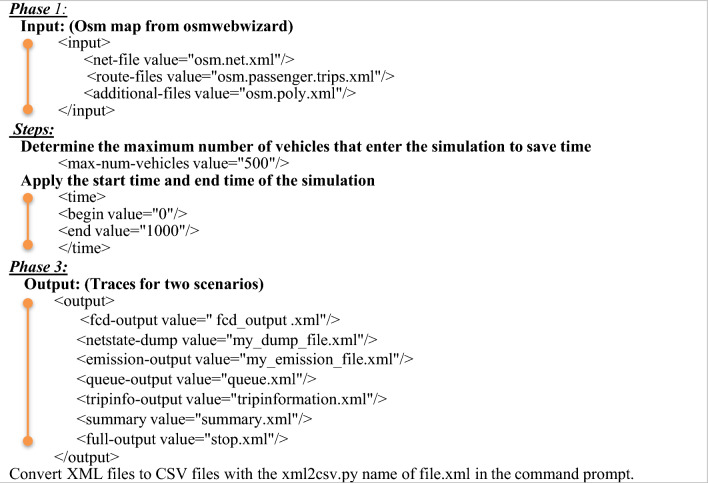
SUMO simulation steps.

## Experimental and results

In this section, we present key results demonstrating the effectiveness of our proposed system using trip information. Table 5 presents a visual comparison of traffic conditions and conflict intensity for Scenarios 1 and 2 at two simulation times (999 and 3599 s), with a 2 s delay and a maximum of 500 vehicles per scenario. Whereas Scenario 1 represents baseline traffic conditions, Scenario 2 represents modified traffic conditions with proposed system improvements.

**Table 5:**
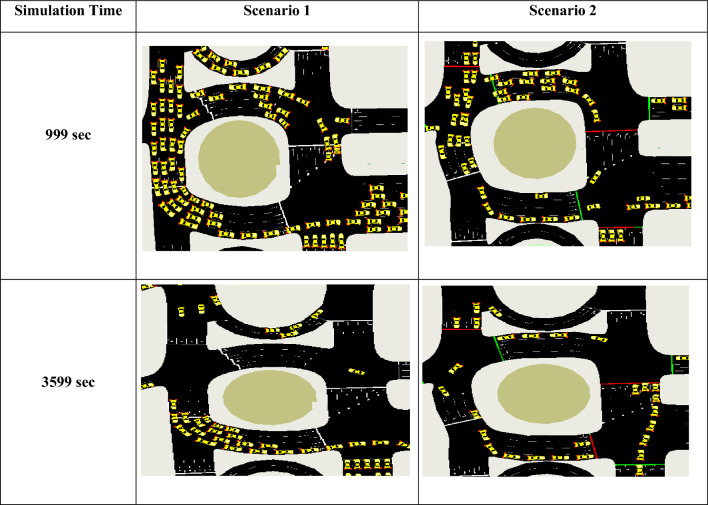
Comparative study illustrated traffic conflict between scenario 1 and scenario 2 after 999 and 3599 sec.

According to the visual analysis, Scenario 1 shows a high vehicle concentration at 999 s, particularly near the intersection entrance and the circulating lanes. Significant congestion and frequent stop-and-go behavior are indicated by closely spaced vehicles and long queues across multiple approaches. The longer trip duration, higher average waiting time, and greater time loss noted for Scenario 1 in the numerical data are all consistent with this visual pattern. Scenario 2, by contrast, exhibits a more orderly vehicle distribution during the same simulation period, with less-dense clusters and smoother intersection traffic. The shorter travel and wait times achieved under Scenario 2 are visually confirmed by the reduced congestion.

The difference between the two situations becomes more pronounced at approximately 3599 s. Even after the prolonged simulation period, Scenario 1 continues to exhibit congestion, with queues persisting. Vehicle congestion may not have subsided entirely, indicating ineffective traffic management, as evidenced by the buildup of vehicles near conflict areas. This finding aligns with the summary data for Scenario 1, which shows a higher mean waiting time and fewer arriving vehicles.

On the other hand, Scenario 2 shows noticeably better traffic conditions at 3599 s. There are significantly fewer vehicle groups, shorter lines, and a more seamless flow of traffic in all directions. The numerical results for shorter wait times, higher throughput, and lower emissions are supported by reduced visual conflict sites, indicating that vehicles experience fewer pauses and delays. Traffic dynamics stabilize with longer simulation times, and Scenario 2 continues to outperform even under constant demand.

Overall, Scenario 2 offers a steadier and more effective traffic operation than Scenario 1. Extending the simulation duration shows that congestion builds up in Scenario 1, but it also highlights how well Scenario 2 manages traffic flow over extended periods. These graphical outputs support the quantitative findings and indicate that Scenario 2 is more appropriate for realistic, long-term traffic conditions.

Tables 6 and 7 compare selected parameters between the first and second scenarios along the entire length of Umm Kulthum Square. In the trip information file, The depart delay (second), which represents the amount of waiting time that the car had before it could begin its trip; arrival (second), when the vehicle arrived at its location; duration (second), which is the amount of time the vehicle required to complete the route, waiting time (second) where the period (planned pauses excluded) during which the vehicle’s speed was less than or equal to 0.1 m/s, time loss (second) represents the time lost as a result of traveling slower than recommended, speed_avg (meter/second) represents the average vehicle speed within the trip. The absolute value of fuel means the fuel consumed during the journey. CO, CO_2_, HC, PMx, NOx, fuel, and the average noise, which represents the noise emitted by the vehicle during the trip.

**Table 6:** Results of the presented system for two scenarios after (999 s).

Simulation time (999 s)	Scenario 1	Scenario 2
Trip Information		
Average (Depart delay(sec))	6.02	5.54
Average (Arrival (sec))	234.71	189.43
Average (Duration (sec))	414.19	349.25
Average (Waiting time)	139.90	100.90
Average (Time loss (sec))	178.18	126.55
Average (Co_abs)	155.37	152.26
Average (Co_2__abs)	2794.72	2745.63
Average (HC_abs	0.87	0.76
Average NOx_abs)	1.92	1.23
Average (PMx_abs)	0.89	0.70
Average (Fuel_abs)	1200.36	875.78
Average noise	59.92	56.86
Queue Output		
Queuing time	128.30	117.64
queueing_length	19.88	17.44
queueing_length _experimental	17.78	14.91
Summary		
Loaded vehicles	1739	1739
Inserted vehicles	762	817
Running vehicles	500	500
Waiting vehicles	971	916
Ended vehicles	262	317
Arrived vehicles	262	317
Mean waiting time	47.01	33.21

**Table 7:** Results of the presented system for two scenarios after (3599 s).

Simulation time (3599 s)	Scenario 1	Scenario 2
Trip Information		
Average (Depart delay(sec))	32.63	7.08
Average (Arrival (sec))	619.22	423.56
Average (Duration (sec))	388.60	246.15
Average (Waiting time)	288.21	143.70
Average (Time loss (sec))	327.88	175.66
Average (Co_abs)	423.16	285.67
Average (Co_2__abs)	4683.61	2968.51
Average (HC_abs	0.93	0.79
Average NOx_abs)	2.92	2.41
Average (PMx_abs)	0.96	0.75
Average (Fuel_abs)	1498.96	916.38
Average noise	76.56	64.94
Queue Output		
Queuing time	136.21	122.06
queueing_length	19.37	17.41
queueing_length _experimental	15.83	11.48
Summary		
Loaded vehicles	6252	6252
inserted vehicles	1172	1215
running vehicles	500	500
waiting vehicles	5078	5035
ended vehicles	672	715
arrived vehicles	672	715
Mean waiting time	199.87	184.43

In the Queue Output file, queuing time (seconds), which represents the entire amount of time that vehicles have to wait in a queue, queuing length (meters), which means the distance between the junction and the last vehicle in the queue, and queuing length.

The Summary file reports the distribution of vehicles across states, providing an overall picture of network performance. The total number of loaded vehicles (all vehicles loaded in the demand), inserted vehicles (vehicles that actually entered the network), running vehicles (vehicles still in motion at the simulation end), waiting vehicles (vehicles halted because of traffic or control), and ended/arrived vehicles (vehicles that successfully finished their trips) are all included. It also reports the mean waiting time, which represents the typical delay experienced by vehicles when stopped. When combined, these metrics indicate the network’s crowding, the effectiveness with which traffic is handled in a given scenario, and whether the simulation time was sufficient for trip completion.

When studying the Trip Information, Summary, and Queue Output files for all inserted vehicles across two traffic control scenarios and two simulation durations, note the differences between the first and second scenarios at each simulation time.

Tables 6 and 7 compare two scenarios at two distinct simulation times (999 s and 3599 s), highlighting how increasing the simulation duration affects traffic performance, queuing behavior, and system capacity. The results clearly show that increasing the simulation period affects numerous numerical indicators but does not improve the system’s overall operating efficiency.

For the Trip Information data, increasing the simulation time from 999 to 3599 s substantially increased most metrics across both scenarios. For example, in Scenario 1, the average arrival time increased from 234.71 s to 619.22 s, while the average waiting time climbed from 139.90 s to 288.21 s. Similarly, time lost went from 178.18 to 327.88 s, while fuel consumption climbed from 1200.36 to 1498.96 units.

Scenario 2 followed the same pattern but performed better, with waiting time growing from 100.90 s to 143.70 s and time lost from 126.55 s to 175.66 s. These numerical increases imply that longer simulation times allow vehicles to remain in the network longer, accumulating delays and emissions rather than increasing traffic flow.

In terms of emissions and environmental impact, a longer simulation time resulted in higher absolute emissions. In Scenario 1, CO₂ emissions rose from (2794.72 to 4683.61) g/km, NOx from (1.92 to 2.92) g/km, and PMx from (0.89 to 0.96) g/km. Scenario 2 showed a similar pattern, with CO₂ levels increasing from (2745.63 to 2968.51) g/km. Furthermore, average noise levels increased significantly, from 59.92 dB to 76.56 dB in Scenario 1 and from 56.86 dB to 64.94 dB in Scenario 2, showing that longer simulation times increase environmental burdens.

The queue output results indicate that queuing behavior remained generally stable over the longer simulation. Queuing time in Scenario 1 increased very slightly from 128.30 s to 136.21 s, but queue length remained nearly constant (19.88 to 19.37 vehicles). Scenario 2 demonstrated comparable stability, with the queue length changing slightly from 17.44 to 17.41 vehicles. This numerical stability implies that congestion is limited by network capacity rather than by simulation time.

The summary results include the most critical findings. As the simulation progressed, the number of loaded vehicles increased dramatically, from 1,739 to 6,252 in both scenarios, indicating a buildup within the system. However, the number of running vehicles remained constant at 500 across all scenarios, indicating that system capacity did not change with increasing simulation time. Furthermore, the number of terminated vehicles was always equal to the number of arriving vehicles —for example, 262 came and 262 terminated in Scenario 1 at 999 s, and 672 arrived and 672 terminated at 3599 s. The same equality was seen in Scenario 2 (317 = 317 and 715 = 715), indicating a perfectly balanced flow.

Based on these numerical results, we conclude that extending the simulation time does not yield any operational benefit. Although longer simulation times result in more loaded and waiting vehicles (increasing from 971 to 5,078 in Scenario 1), they do not increase throughput or the number of completed trips because the number of running vehicles remains constant. The number of ended vehicles is always equal to the number of arrived vehicles.

**Fig. 6:**
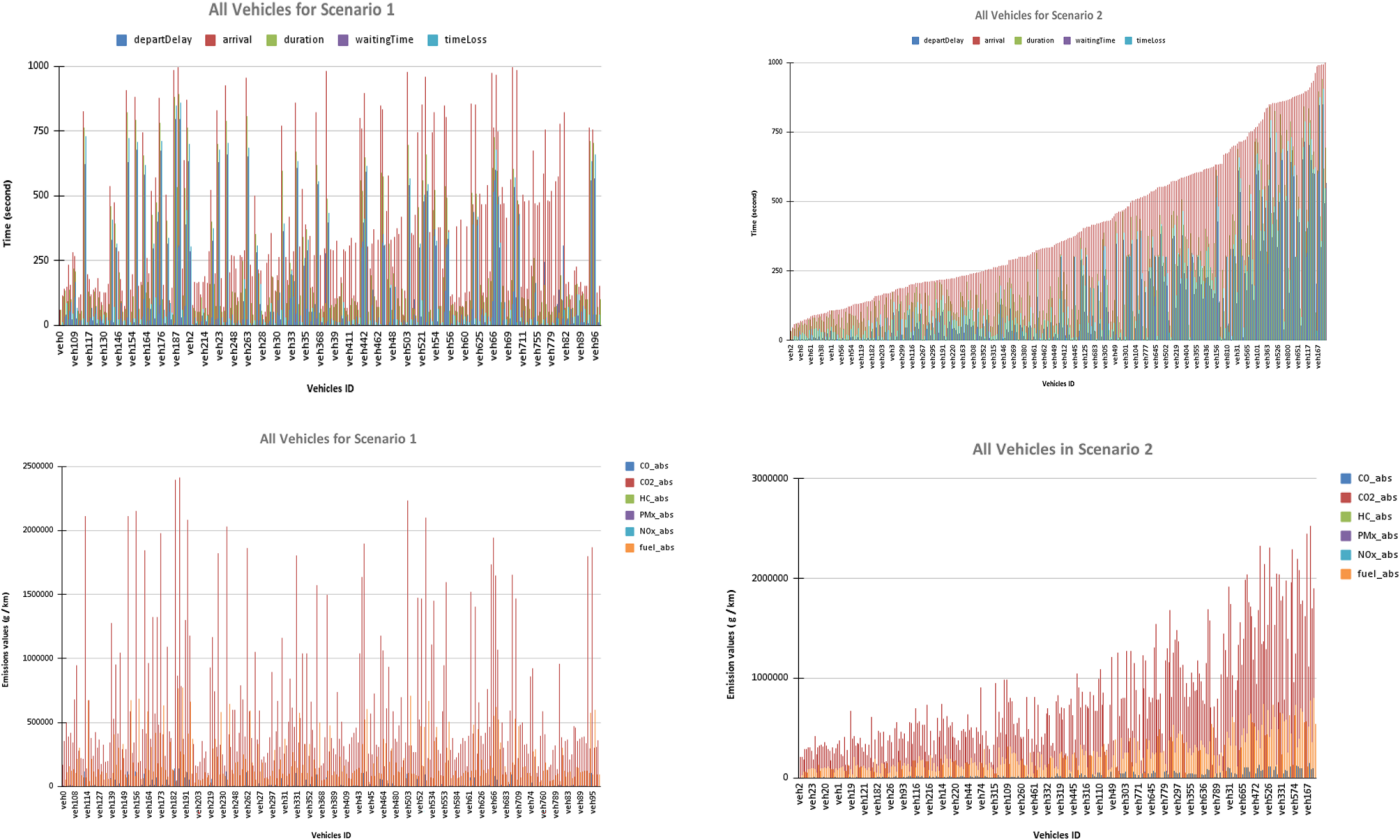
Proposed A System for Some Parameters between the First and the Second Scenarios for All Vehicles Inserted into the Simulation after 999 s.

Figure 6 compares the proposed system’s performance across key factors in the first and second scenarios for all vehicles included in the simulation. We divide the figure into four subplots, each showing a different metric over time, using data from Scenario 1 on the left and Scenario 2 on the right. Taking into account that the vehicles entering the first scenario were 262, which arrived from 500 cars running, and that in the other scenario, 317 vehicles came from 500 vehicles running.

- Top Row: Represents Departure Delay, Arrival Time, Waiting Time, Time Loss, and Duration for all vehicles.
- Y-axis: Time in seconds (e.g., 0 to 1200 s).
- X-axis: number of vehicles.
- Scenario 1 (Left) shows significant fluctuations in departure delay (light blue), arrival (orange), waiting time (yellow), time loss (dark blue), and duration (grey). High peaks indicate substantial delays and waiting times, suggesting congestion and inefficiencies.
- Scenario 2 (Right) displays a more stable trend, with a noticeable increase in arrival (orange) and reduced peaks in waiting time (yellow) and time loss (dark blue), indicating improved traffic flow and decreasing delays after modifications.
- Bottom Row: represents emission levels (CO, CO₂, HC, PMx, NOx, Fuel) for all vehicles
- X-axis: number of vehicles.
- Y-axis: Emission levels or fuel consumption (milligrams)
- Scenario 1 (Left) includes high and erratic bars for CO (light blue), CO₂ (orange), HC (grey), PMx (yellow), NOx (dark blue), and fuel (green), indicating significant emissions and fuel use due to prolonged idling and congestion.
- Scenario 2 (Right) includes lower and more consistent emission levels across all categories, with a gradual increase rather than sharp spikes, suggesting a reduction in emissions and fuel consumption due to decreased waiting times and smoother traffic movement.

As shown in Fig. 6, the proposed system in Scenario 2 effectively mitigates traffic conflicts. It improves efficiency relative to Scenario 1, as indicated by lower metrics for delays, waiting times, and emissions throughout the simulation period.

## Conclusion

This study assessed the effectiveness of urban traffic management strategies at Umm Kulthum Square in Mansoura, Egypt, using the Simulation of Urban Mobility (SUMO) software. By comparing existing traffic conditions with an optimized scenario, the results demonstrated that improvements in lane configurations, turning movements, and traffic signal timing can significantly enhance traffic performance at congested urban intersections. The optimized scenario achieved notable reductions in departure delay, travel duration, waiting time, time loss, queuing time, and queue lengths, along with an increase in completed trips, indicating smoother traffic flow. In addition to operational improvements, substantial environmental benefits were observed, including reduced emissions of CO₂, CO, HC, NOx, and PMx, as well as lower fuel consumption and noise levels. These findings confirm that simulation-based traffic optimization is a practical and cost-effective approach for improving traffic operations in dense urban areas, particularly in developing cities such as Mansoura. The proposed methodology provides a data-driven foundation for traffic planners and decision-makers to implement effective traffic signal optimization and intersection design strategies. It supports future integration of adaptive and sustainable traffic control solutions. This will help us better understand how proposed traffic flow modifications may affect the region. Based on the simulation results, Traffic signal priority entails adjusting phase durations and sequencing at critical intersections to reduce wait times and increase traffic flow during peak hours. Intersection design enhancements include modifying lane connections and turning actions to reduce vehicle conflicts and congestion. Time-based traffic management solutions involve implementing dynamic signal timing that responds to real-time traffic conditions, underscoring how simulation results can inform effective traffic policies for Umm Kulthum Square.

## Future work

The research suggests that future transportation policy should prioritize the implementation of adaptive traffic control systems to reduce travel and waiting times, as well as emissions. Real-time, AI-driven traffic light systems should be developed to dynamically respond to traffic conditions in congested areas, thereby enabling more efficient traffic management. Egypt should also invest in innovative country initiatives, particularly in deploying ITS for traffic monitoring, predictive analytics for congestion management, and real-time rerouting via user navigation apps. These policy directions align with Egypt’s urban development goals and can be tailored for similar cities facing traffic congestion challenges. Future research could expand on this work by applying the simulation framework across different intersections and integrating socio-economic variables to achieve more inclusive policy outcomes.

## Data Availability

The data supporting the findings of this study are provided in this article.
